# CT imaging findings of invasive pulmonary fungal infections in hemato-oncologic children

**DOI:** 10.1186/s13244-024-01871-w

**Published:** 2024-12-12

**Authors:** Leonor Alamo, Francesco Ceppi, Estelle Tenisch, Catherine Beigelman-Aubry

**Affiliations:** 1 Department of Radiology, Lausanne University Hospital (CHUV), Lausanne, Switzerland; 2 University of Lausanne (UNIL), Lausanne, Switzerland; 3 Pediatric Hematology-Oncology Unit, Department of Woman-Mother-Child, Lausanne University Hospital (CHUV), Lausanne, Switzerland

**Keywords:** Pulmonary invasive fungal infections, Aspergillosis, Mucormycosis

## Abstract

**Abstract:**

Hemato-oncologic children form a heterogeneous group with a wide spectrum of ages, malignancy types, and immunosuppression grades during the different phases of their treatment. Immunosuppression is caused by multiple factors, including the malignancy itself, bone marrow suppression secondary to therapy, and wide use of steroids and antibiotics, among others. At the same time, the risk of infections in these patients remains high because of prolonged hospitalizations or the need for long-timing implanted devices between other features. In this context, a pulmonary fungal infection can rapidly turn into a life-threatening condition that requires early diagnosis and appropriate management. This pictorial essay illustrates the main imaging findings detected in chest computed tomography examinations performed in pediatric hemato-oncologic patients with proven pulmonary invasive fungal infections caused by *Candida*, *Aspergillus*, or *Mucor*. In addition, it describes useful clues for limiting differential diagnoses, reviews the literature on pediatric patients, and compares imaging findings in adults and children.

**Critical relevance statement:**

The main fungal pathogens causing invasive fungal infections (IFI) in hemato-oncologic children are *Candida*, *Aspergillus*, and *Mucor*. This review describes the most frequently affected organs and the most common imaging findings detected in chest CT exams in children with pulmonary IFI.

**Key Points:**

- To review the main computed tomography imaging findings suggesting pulmonary invasive fungal infection (IFI) in hemato-oncologic children.
- To describe differences between pediatric and adult patients with proven pulmonary IFI.
- To provide useful clues for limiting the differential diagnosis of pulmonary IFI in pediatric patients.

**Graphical Abstract:**

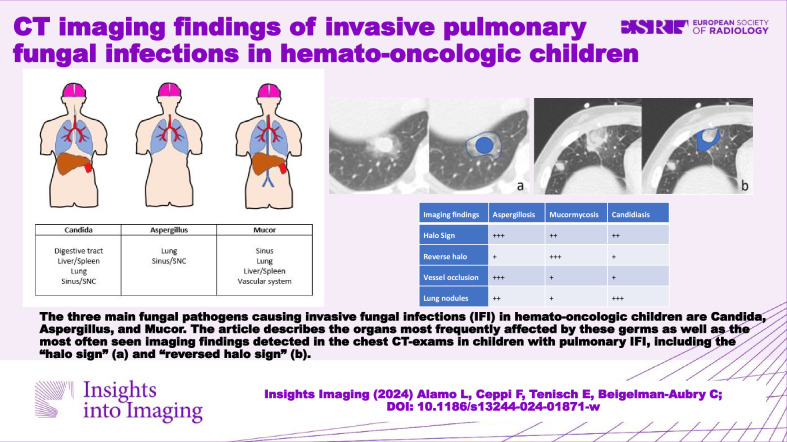

## Introduction

Hemato-oncologic children form a heterogeneous group of patients with a wide spectrum of ages, malignancies, and grades of immunosuppression during the disease [1–3]. Alteration of the immune system is multifactorial and depends not only on the malignancy itself but also on the aggressive required therapies or the wide use of steroids and broad-spectrum antibiotics, among others [4, 5]. Moreover, the risk of opportunistic infections during treatment remains high because of iterative prolonged hospitalizations or long-term use of invasive devices between other features. Particularly sensitive are children treated with hematopoietic stem cell transplantation (HSCT), with prolonged severe neutropenia associated with the procedure [1].

**Fig. 1:**
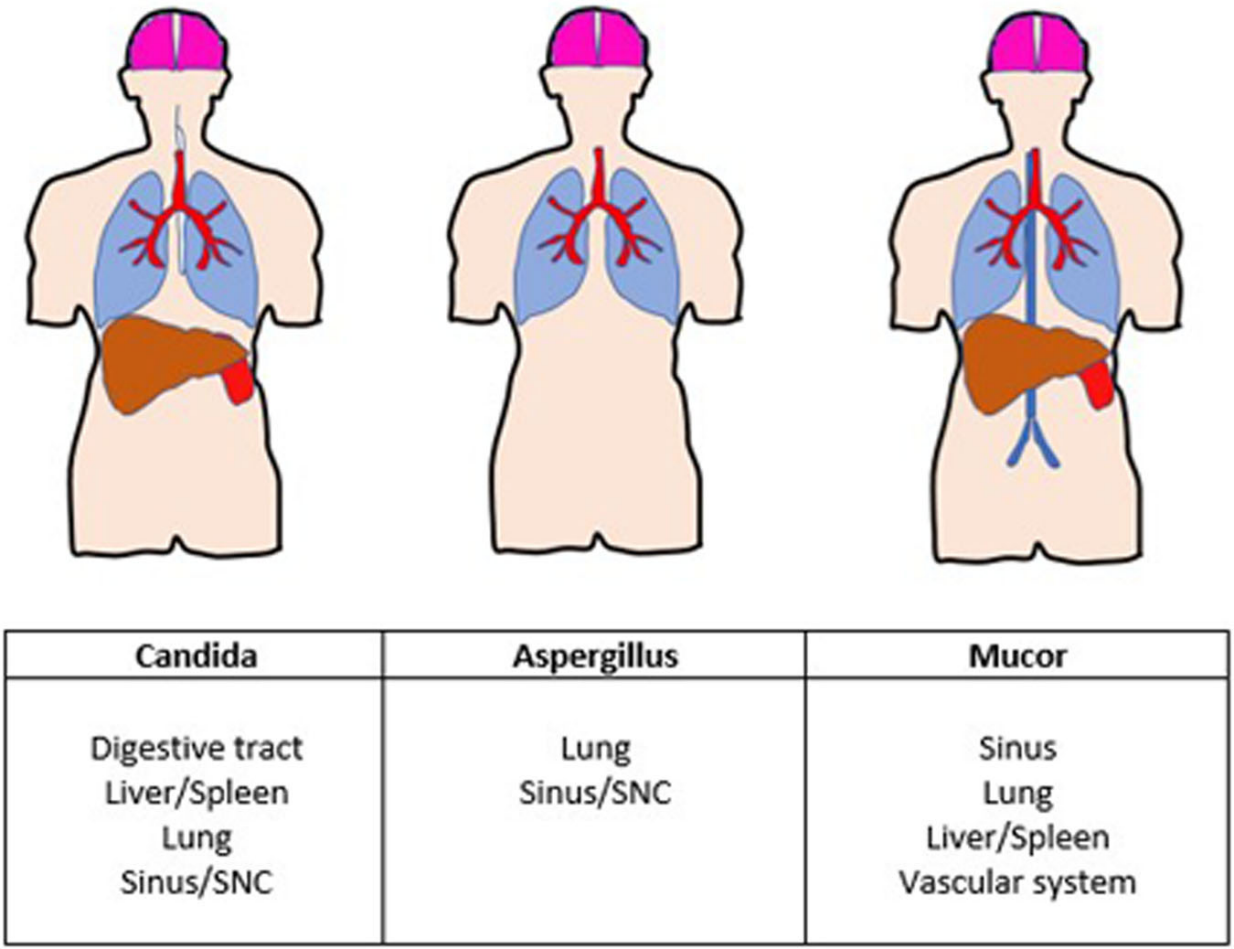
Most frequently affected organs in *Candida*, *Aspergillus* and *Mucor* invasive fungal infections

Invasive fungal infections (IFI) are often detected in these children. Two recent studies revealed an identical 24% incidence in children with acute lymphoblastic leukemia (ALL) [2] and in pediatric hemato-oncologic patients treated with HSCT [5]. In this context, IFI can rapidly develop into a life-threatening condition that requires early diagnosis and appropriate therapy [2, 5, 6]. The three main pathogens responsible for IFI worldwide are *Candida*, *Aspergillus* and *Mucor*. The paranasal sinuses and respiratory tract are by far the most frequently affected organs, with 60–80% of cases [7], followed by the liver, spleen, kidneys, eyes, and central nervous system [7, 8] (Fig. 1 and Table 1) [9].

**Table 1:** Most frequently detected invasive fungal pathogens for each system (Modified from ref. [9])

System	Associated fungal pathogen
CNS	Meningitis: *Candida* Abscess: *Aspergillus*, *Mucorales*
Paranasal sinuses	*Mucorales*, *Aspergillus*
Respiratory system	*Aspergillus*, *Candida*, *Mucorales*
Gastrointestinal	*Candida*
Genitourinary	*Candida*, *Aspergillus*
Cardiac	*Candida*, *Aspergillus*

*Candida*, *Aspergillus*, and *Mucor* are also the three most common respiratory fungal infections detected [10–14]. Hemato-oncologic children account for approximately 10% of all invasive aspergillosis (IA) diagnosed in the USA [10], with ALL as the most frequent underlying hematologic pathology [15]. *Mucor*’s infections are increasing and remain associated with high rates of severe morbidity and mortality [13]. Differentiating between invasive Aspergillosis (IA) and Mucormycosis remains crucial in a symptomatic child because the preferred antifungal therapy for *Aspergillus*—voriconazole—is not effective against *Mucor* [6], and a delay in diagnosis may lead to a rush *Mucor* dissemination with catastrophic consequences [16–18].

This article describes the main imaging findings observed in chest computed tomography (CT) examinations performed in hemato-oncologic children with proven pulmonary IFI by *Candida*, *Aspergillus*, or *Mucor*. It reviews the literature concerning pediatric patients and discusses the criteria for limiting the differential diagnosis. Clinical data and images were obtained from patients diagnosed and treated at a tertiary center for pediatric oncology in Switzerland. The local Institutional Review Boards approved the manuscript for publication.

### Respiratory invasive fungal infections: general points

In a hemato-oncologic child, the development of fever and/or nonspecific respiratory symptoms should rapidly suggest possible pulmonary IFI. To facilitate diagnosis, patients are often monitored using serum galactomannan and/or blood polymerase chain reaction [11, 19]. Plain chest radiography is usually the initial imaging modality performed, but early chest contrast-enhanced CT remains crucial for obtaining accurate information about the possible etiology, extent, and severity of the infection [3]. CT may also indicate the best location for bronchoalveolar lavage or the most appropriate nodule for biopsy if required. Low-dose follow-up CT examinations may assess patient response to therapy [20, 21].

**Fig. 2:**
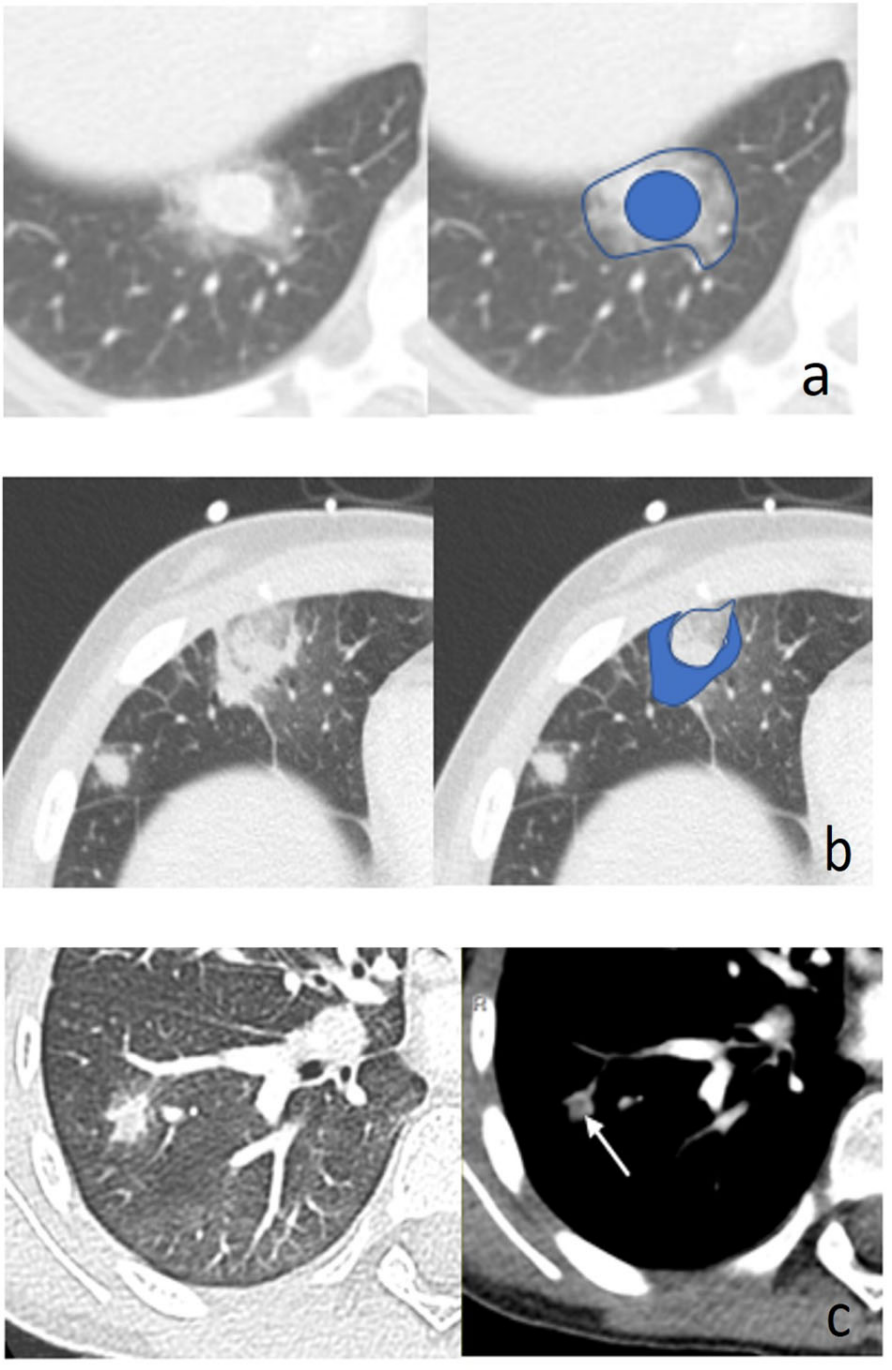
Chest computed tomography (CT) imaging signs suggesting invasive fungal infection (IFI). Axial chest CT images show the “halo sign” (**a**), consisting of a solid lung nodule (in blue) surrounded by a ring of ground glass in the right lower lobe. The “reversed halo sign” (**b**) appears as an area of ground-glass opacity surrounded by an almost complete ring of pulmonary consolidation (in blue) in the middle lobe. The “hypodense sign” (**c**) shows central necrosis (arrow) in a solid nodule in the right lower lobe

Enlarged lymph nodes and isolated ground-glass opacities are atypical of fungal disease. On the other hand, some CT imaging findings may suggest fungal infection because they are more commonly associated with these pathogens than with other infectious causes [3, 22, 23] (Fig. 2). However, it is important to remember that none of these signs are specific to either a fungal infection or specific fungus [3, 22, 23]. The “halo sign” (Fig. 2a) consists of a solid, varied-sized nodule surrounded by a ring of parenchymal ground-glass attenuation [14]. It is reported as the most common CT finding in immunosuppressed patients with IFI and usually considered an early image of the infection [14]. It was also the most frequently detected sign (67.5%) in a series of 40 hemato-oncologic children with confirmed IFI [7]. Although classically described in invasive Aspergillosis (IA) [1, 7, 22], it is occasionally detected in Mucormycosis and Fusariosis, in infections by *Pseudomonas aeruginosa*, *Mycobacterium tuberculosis* and *Herpes virus*; in granulomatosis with polyangiitis, and in Kaposi’s sarcoma [3, 16, 22, 23]. The “reverse halo” consists of a focal rounded area of ground-glass opacity surrounded by a crescent or complete ring of parenchymal consolidation [6] (Fig. 2b). It is often observed in IFI, more commonly in Mucormycosis (54%) than in IA (6%), and in some cases of *Pneumocystis Jiroveci* pneumonia, lipoid pneumonia, pulmonary vasculitis, tuberculosis or sarcoidosis [22, 24].

The “hypodense sign” represents internal necrosis in a solid nodule (Fig. 2c), whereas the “air-crescent sign” indicates eccentric cavitation of a previous solid nodule. It usually appears during recovery from neutropenia, 2–3 weeks after the onset of the disease [14]. In addition to IFI, these signs can be observed in tuberculosis and some lung abscesses [22]. Finally, the “vessel occlusion sign” (VOS) reflects the occlusion of small pulmonary arteries by fungal hyphae, which is visible as the interruption of a vessel at the border of a suspected lesion [22, 25]. It is strongly associated with IA but is also occasionally observed in Mucormycosis [23, 25].

In 2008, the European Organization for Research and Treatment of Cancer (EORTC) classified the “halo sign”; the “air-crescent sign” and lung cavitations as typical IFI findings [26]. A revision in 2020 has added lung consolidations and nodules without a halo as indicative patterns [27]. Although these patterns were mainly described in adults, a recent retrospective series in children with proven IFI revealed a 97.5% incidence of typical CT findings when the authors applied the 2020 guidelines’ version [7]. Imaging findings seem to be more variable in children than in adults and may even show differences in children of different ages [3, 7]. “Halo sign” and cavitations are more often observed in adults than in children, and in older children and adolescents than in younger patients, probably because of differences in the host immune response [3, 7]. Table 2 describes the most often pulmonary patterns observed for each fungal pathogen.

**Table 2:** Frequency of the main chest CE-CT imaging signs detected in pulmonary aspergillosis, mucormycosis and candidiasis [1, 6, 7, 13, 15, 21, 22, 24, 27, 29]

Imaging findings	Aspergillosis	Mucormycosis	Candidiasis
Halo sign	+++	++	++
Reverse halo	+	+++	+
Vessel occlusion	+++	+	+
Lung nodules	++	+	+++

### Peculiarities according to fungal pathogens in pediatric IFI

#### Invasive candidiasis

Pulmonary invasive candidiasis caused by the aspiration of *Candida*-contaminated oropharyngeal secretions is rare and occurs mainly in neonates [4, 16, 28, 29]. The most common form of pulmonary dissemination in hemato-oncologic children is hematogenous after a previous cutaneous or gastrointestinal infection or following venous catheter contamination by *Candida* [3, 16]. Multifocal involvement with tiny hepatic, splenic, and/or renal abscesses is frequently observed and helps in the diagnosis. The most common CT findings (88–95%) in adults are multiple bilateral, different-sized but mostly small (3–30 mm) lung nodules in a miliary pattern [4, 28, 30], with a centrilobular (52%) or random (48%) distribution pattern [30]. Other nonspecific imaging findings include ground-glass opacities (35%), “halo sign” (33%), alveolar consolidations, and tree-in-bud opacities. Cavitations are uncommon (only 4%) and a “reverse halo sign” is rarely detected [28, 30].

**Fig. 3:**
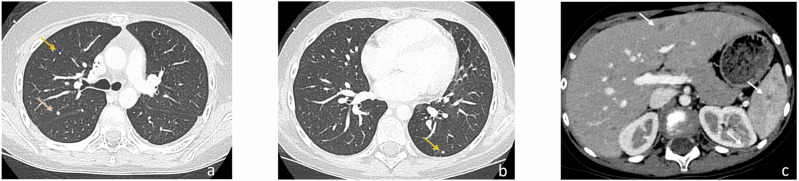
Multiple lung nodules in proven pulmonary candidiasis in an 8-year-old girl with acute lymphoblastic leukemia (ALL). Note the multiple, bilateral, small-sized, randomly distributed pulmonary nodules in the middle lobe (arrows **a**) and left lower lobe (**b**). The detection of additional renal and hepatosplenic involvement (arrows **c**) facilitates diagnosis

**Fig. 4:**
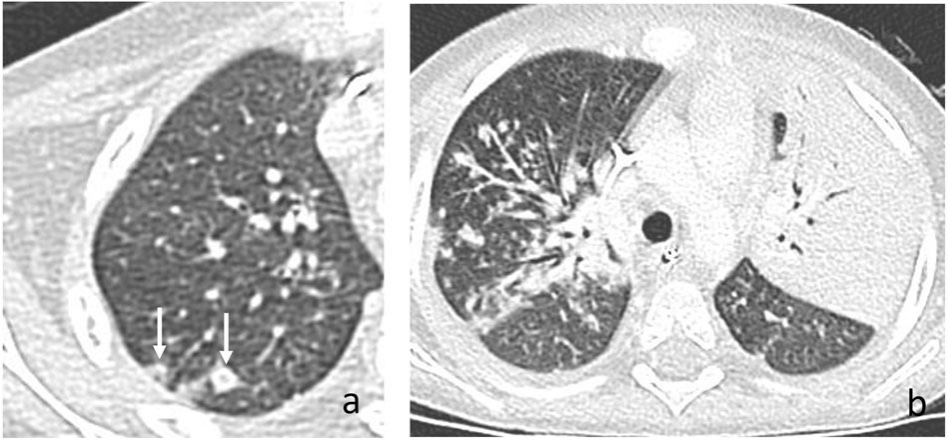
Proven pulmonary candidiasis in a 3-year-old boy with Burkitt’s lymphoma. The patient developed fever and increasing respiratory symptoms while in agranulocytosis under chemotherapy. Initial chest contrast-enhanced computed tomography (CE-CT) images on lung window (**a**) show a “reverse halo sign” in the right upper lobe (arrows). Follow-up CE-CT exam (**b**) one week later revealed rapid progression of the illness with multiple, varied-sized pulmonary nodules. A new extensive alveolar consolidation is detected in the left upper lobe

**Fig. 5:**
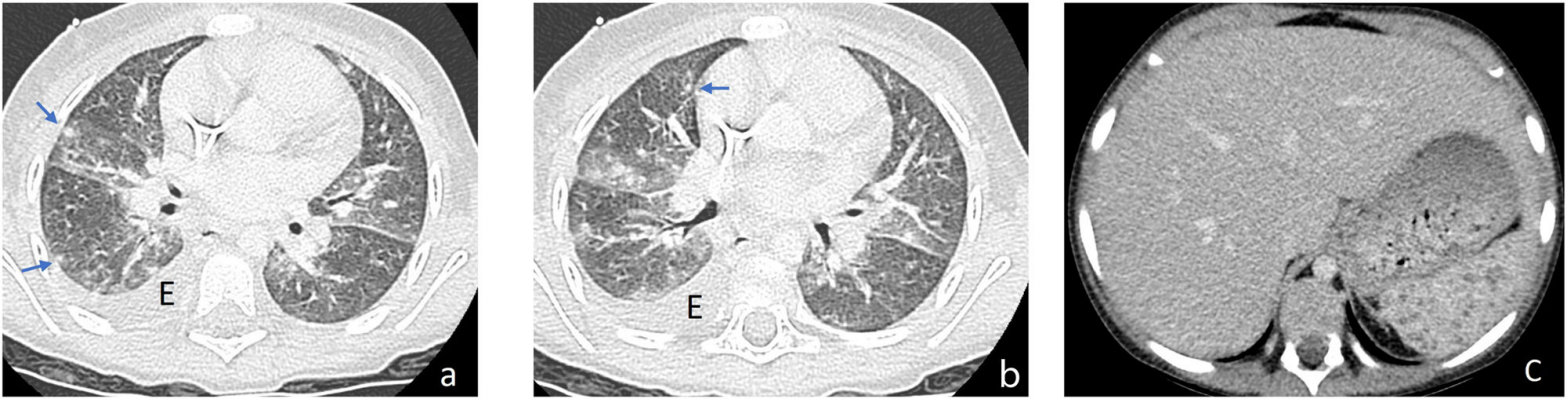
Proven invasive candidiasis in a 4-year-old boy with pre-B cell acute lymphoblastic leukemia (ALL) under chemotherapy. The patient developed non-productive cough, fever, and skin nodules in agranulocytosis. Axial chest contrast-enhanced computed tomography (CE-CT) images in lung window (**a**, **b**) show multifocal areas of ground-glass infiltrates, multiple small-sized lung nodules (blue arrows) and moderate right pleural effusion (E). Although the findings were nonspecific, the development of synchronic skin nodules and splenic lesions (**c**) highly suggested disseminated candidiasis, confirmed by biopsies of the cutaneous nodules and buccal mucosa

There are limited descriptions of the radiological appearance of pulmonary candidiasis in immunosuppressed pediatric patients. The miliary pattern is occasionally observed during the initial phase of the infection (Fig. 3), but later findings may differ and be challenging [3, 31]. A review of the autopsy records of 14 infants with proven pulmonary candidiasis identified three different histological patterns: embolic (arterial invasive), disseminated (capillary invasive), and bronchopulmonary. However, the authors concluded that later findings were mixed with coexistent lung infarctions or pneumonia caused by other germs [3]. A recent series in hemato-oncologic children with proven lung Candidiasis identified nonspecific findings, including multiple nodules, bronchial wall thickening, alveolar consolidations, ground-glass attenuation, and pleural effusion [7]. In our experience “halo-” and “reverse halo sign” are also occasionally observed (Figs. 4, 5). Synchronous detection of hepatic and/or splenic lesions helps identify the cause of infection.

#### Invasive pulmonary aspergillosis

*Aspergillus fumigatus* is by far the most frequently reported pulmonary fungal pathogen in hemato-oncologic children [7]. Patients with acute leukemia treated with HSCT, high doses of corticosteroids or other immunosuppressive therapies are particularly sensitive [15]. The most frequently observed manifestation is invasive pulmonary aspergillosis (IA), which includes the broncho-invasive and angio-invasive forms. Both forms may coexist in the same patient, making it difficult to differentiate them from each other [4, 14, 15]. The presentation observed in immunocompetent patients, such as aspergilloma, allergic bronchopulmonary, chronic cavitary, or necrotizing aspergillosis, remains rare in these children [15].

Airway-invasive aspergillosis is difficult to diagnose because the clinical symptoms (stridor, wheezing) and imaging abnormalities remain subtle. It can appear as a clinically acute tracheobronchitis, with tracheal or bronchial wall thickening on CT [32]. If it progresses, bronchiolitis develops, with mainly centrilobular nodules and branching opacities with a patchy “tree-in-bud” appearance [4]. Bronchopneumonia results in nonspecific peribronchial densities and different-sized consolidation areas, but lobar consolidations remain rare [29, 33].

Angio-invasive aspergillosis is the most common form of IA. It is a life-threatening condition, with a mortality rate of 50–85% in adults [25, 34]. Infection is particularly dangerous in severely neutropenic patients treated with HSCT, with only a 25.4% one-year survival rate reported after proven infection [35]. In adults, the most common and characteristic initial CT sign is the coexistence of different-sized lung nodules with “halo sign” and patchy areas of ground-glass attenuation [3, 16]. Nodules are mostly centrilobular (96%) located versus randomly (4%) distributed [4, 29]. The “reverse halo” sign is rare in the initial phase of infection (6%) [24] but can be observed later. Histologically, the infection is characterized by the invasion and occlusion of small pulmonary arteries by fungal hyphae, causing the “hypodense-” and the “VOS sign” [4, 25] (Fig. 2). Recent studies suggest that the “VOS sign” is the strongest diagnostic radiological finding for IA and is more sensitive and specific than the “halo sign” [23, 25, 35]. In cases of progression, invasion of major pulmonary arteries results in thrombosis and distal infarction with multiple, different-sized and pleura-based wedge-shaped lung consolidation areas or hemorrhagic infarct [29].

**Fig. 6:**
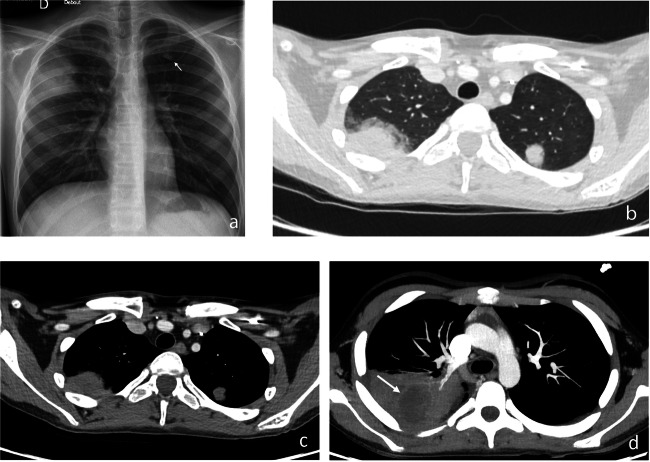
Proven invasive pulmonary aspergillosis in a 16-year-old boy with acute myeloblastic leukemia (AML) under chemotherapy. Chest X-ray (**a**) performed because of increasing thoracic pain after minor trauma evidences an extensive lung infiltrate with ill borders in the right upper lobe and a nodule below the chondro-costal junction of the 1st left rib (arrow). Axial chest CE-CT image in pulmonary (**b**) and mediastinal (**c**) window reveals that both nodules are partially surrounded by ground-glass opacity (“halo sign”). Diagnosis was confirmed by bronchoalveolar lavage. Maximum intensity projection (MIP) images of the follow-up chest CE-CT examination (**d**) in mediastinal window performed 4 days later show increasing extent of the right upper lobe mass, now with central necrosis

**Fig. 7:**
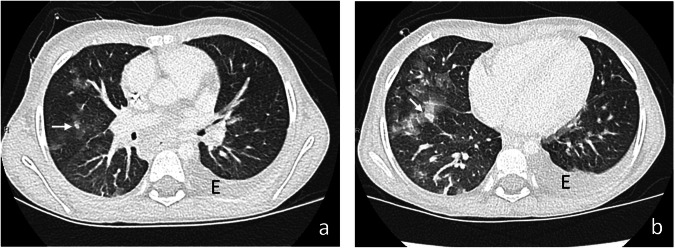
Proven invasive pulmonary aspergillosis in a 4-year-old girl with acute myeloblastic leukemia (AML) under chemotherapy. Chest CE-CT scan performed because of increasing cough and fever evidenced multiple patchy areas of ground-glass attenuation and pulmonary nodules (white arrows **a** and **b**) surrounded by ground-glass opacity (“halo sign”). Note also moderate left pleural effusion (E). Diagnosis was confirmed by bronchoalveolar lavage

**Fig. 8:**
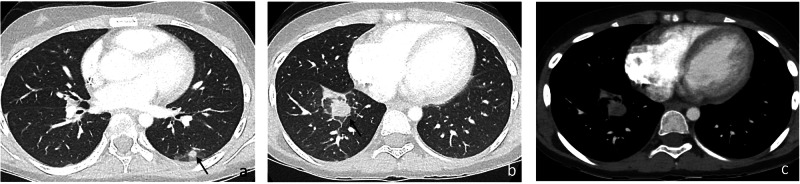
Initial invasive pulmonary aspergillosis in a 11-year-old girl with acute lymphoblastic leukemia (ALL) pre-B. Axial images of chest CE-CT scan in lung (**a**, **b**) and mediastinal (**c**) window show bilateral lung nodules with “halo sign” (arrows). Note the various size on the nodules and the central “hypodense sign” in the largest one (**c**)

In a child with appropriate clinical settings, the initial detection of multiple lung nodules with “halo” should be considered highly suggestive of IA [3, 23] (Figs. 6–8). However, CT findings in children are more variable and often nonspecific [16, 36]. Cavitation, “halo-” and “air-crescent” signs are less frequently detected than in adults. A large multicenter retrospective review of 139 children with proven IA [36] revealed aspecific lung nodules without “halo” as the most frequent finding (34.6%). Cavitation was observed in 24.5% of cases, most commonly in adolescents, and “halo-” and “air-crescent” signs were only detected in 11% and 2.2% of cases, respectively [36].

#### Mucormycosis

The most frequently reported pathogens of the order *Mucorales* are *Rhizopus* and *Mucor* species. The incidence of pulmonary Mucormycosis seems to be increasing, probably because of a larger population at risk, increased use of immunosuppressors and higher survival rates of hematologic malignancies [37]. Prognosis in patients with hematologic malignancies or after HSCT remains poor, with a reported mortality rate of 6–33% that increases to 30–90% in case of dissemination [13, 16]. The lungs are the second most commonly involved organ after rhinocerebral invasion (Fig. 1 and Table 1).

In adults, early chest CE-CT scans often reveal unifocal alveolar consolidation related to pneumonia, mostly located in the upper lobes, that rapidly disseminates by vascular invasion, with a common “reverse halo sign”. Extensive parenchymal necrosis leads to prompt abscess formation and cavitation, which is observed in as many as 40% of cases [4, 38]. In cases of late diagnosis or inadequate response to therapy, there is a rush extensive invasion of contiguous structures such as the chest wall, spine, aorta, pericardium and/or diaphragm [4, 37]. Other findings include progressive lobar or multilobar consolidation (66%), solitary or multiple lung nodules (16%) and masses (25%). The “reverse halo sign” is detected in 65% of patients [24] and can suggest diagnosis, especially in case of concomitant sinusitis [3], whereas the “halo sign” appears in 19–53% of cases [17]. The “air-crescent sign”, rare in other IFIs, is here reported in 13–54% of cases [4, 38]. In CE-CT examinations, abrupt termination of the pulmonary artery branches may result in arterial pseudoaneurysms with hemoptysis [39]. Enlarged lymph nodes, pleural thickening, and pleural effusion are more frequently observed in Mucormycosis than in other fungal pneumonia [37].

**Fig. 9:**
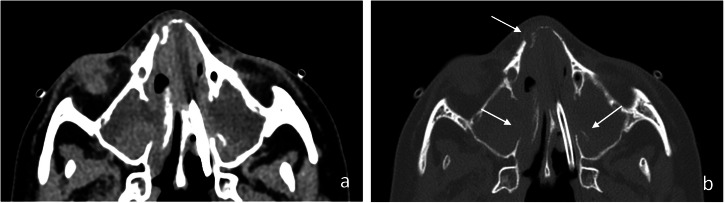
Proven invasive sinus mucormycosis in a 10-year-old girl after hematopoietic stem cell transplantation therapy. Axial CE-CT images of paranasal sinuses in soft tissue (**a**) and bone window (**b**) show bilateral complete opacification of the maxillary sinus with heterogeneous, partially hyperintense content. Note extensive bone erosion and lytic destruction of the nasal bones (arrows, **b**)

**Fig. 10:**
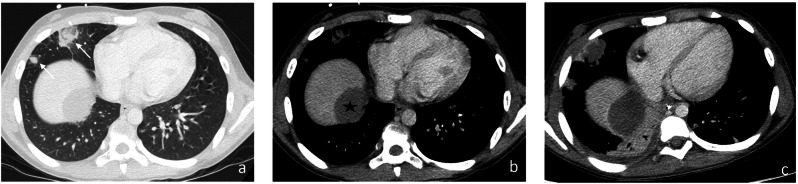
Proven pulmonary mucormycosis in a 9-year-old girl with acute myeloblastic leukemia (AML) under chemotherapy. Axial images in lung (**a**) and mediastinal (**b**) window evidenced “reverse halo sign” in the right middle lobe and a small peripheral lung nodule with “halo sign” (arrows, **a**). There was also a voluminous necrotic liver abscess (star, **b**). Follow-up exam 2 days later (**c**) showed rapid increase in size and extensive necrosis of lung nodules, infiltration of the adjacent pleura, development of a new alveolar consolidation in the right lower lobe with mild pleural effusion and new lung nodules in the left lower lobe. The size of the liver abscess increased with diaphragmatic infiltration (not shown). The patient died some days later after unsuccessful therapy

**Fig. 11:**
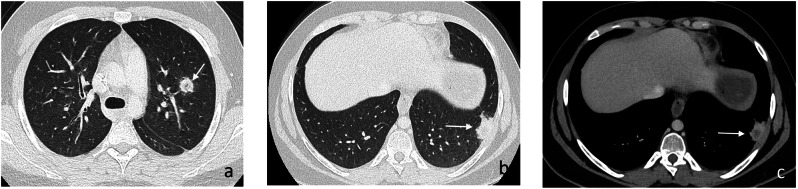
Proven pulmonary mucormycosis in a 17-year-old boy in agranulocytosis by acute myeloblastic leukemia (AML) under chemotherapy. Axial CE-CT images in lung (**a**, **b**) and mediastinal window (**c**) show a lung nodule with reversed halo sign in the left upper lobe LUL (arrows, **a**) and a subpleural located lesion in the posterior left lower lobe with “hypodense sign” (arrows, **b**, **c**) indicating central necrosis and probably the onset of cavitation. Note focal pleural infiltration

Epidemiological data on *Mucor*-induced pulmonary infections in children are scarce. Patients with ALL are especially sensitive during induction therapy [37]. In a child under voriconazole prophylaxis, clinical symptoms and CT findings of sinusitis, such as extensive sinus opacification, deep fascial plane obliteration, and bony erosion, should be seriously considered highly suggestive of diagnosis [3, 37] (Fig. 9). A multicentric series of children with proven invasive Mucormycosis revealed concomitant sinusitis in 14 of 15 patients (93.3%) [12]. The few published pediatric cases of proven infection describe an initially focal, usually segmental consolidation often associated with pleural effusion that rapidly increased in size and developed extensive parenchymal necrosis and cavitation [12, 40–42] (Figs. 10, 11). Arterial pseudoaneurysms with hemoptysis after vascular occlusion, although rare, have also been reported in children [42]. Hematogenous dissemination occurs in 18–50% of cases, and mortality rates oscillate between 33 and 56% of cases [41].

## Conclusion

Immunosuppressed hemato-oncology children have an increased risk of pulmonary IFI during their disease, with still high rates of severe morbidity and mortality. Early chest computed tomography is crucial for orienting diagnosis. Imaging findings such as the “halo-”- and the “reverse halo sign” strongly suggest fungal pneumonia but remain nonspecific.

The most frequently detected CT finding is the presence of multiple, nonspecific lung nodules. Multiple bilateral, small-sized, centrilobular, or randomly distributed nodules without “halo” suggest candidiasis, especially if there are concomitant tiny hepatic, splenic, and/or renal lesions. Differentiating Aspergillosis from Mucormycosis remains challenging. Multiple, mostly centrilobular located nodules with “halo” favor Aspergillosis, whereas concurrent sinusitis, previous voriconazole therapy, pleural effusion, “reverse halo sign” and rush invasion of contiguous structures favor Mucormycosis.

## Data Availability

All clinical data and chest CT images are available for the authors following the conditions for access applied by the corresponding Ethics Committee (CER-VD).

## Abbreviations

ALL: Acute lymphoblastic leukemia
CT: Computed tomography
HSCT: Hematopoietic stem cell transplantation
IA: Invasive aspergillosis
IFI: Invasive fungal infection
VOS: Vessel occlusion sign
